# The impact of first COVID-19 peak on patient referrals to Liaison Psychiatry Service and staff perception about service provision in Birmingham and Solihull Mental Health Trust Birmingham - a service evaluation project

**DOI:** 10.1192/bjo.2021.865

**Published:** 2021-06-18

**Authors:** Saima Jehanzeb, Muhammad Suleman, Ella Tumelty, Joanne Okusanya, Laxsan Karunanithy, Lucretia Thomas, Rebecca Marshall, Dhruba Bagchi, Mahnaz Hashmi

**Affiliations:** 1 Birmingham and Solihull Mental Health NHS Foundation Trust; 2 Birmingham Community Healthcare NHS Foundation Trust; 3 Medical School, College of Medical and Dental Sciences, University of Birmingham

## Abstract

**Aims:**

Based on recommendations from the Royal College of Psychiatrists, this project aimed to evaluate the impact of the first peak of the COVID-19 pandemic on referral patterns to the Queen Elizabeth Hospital Birmingham (QEHB) Liaison Psychiatry (LP) service. Additionally, we aimed to explore staff experiences in LP services across Birmingham and Solihull Mental Health Trust (BSMHFT) in order to generate Trust recommendations promoting optimal healthcare provision amidst the on-going pandemic.

**Method:**

A mixed method service evaluation was conducted using quantitative and qualitative analysis. Quantitative methods involved reviewing referrals made to the QEHB LP service from March to June 2020, compared with the equivalent time period in 2019. Data were retrospectively extracted from the electronic clinical databases RIO and PICS, and subsequently analysed using Microsoft Office. The number of, and reasons for referrals to LP were identified, whilst focus groups were conducted to explore the subjective experiences of staff working across BSMHFT LP services.

**Result:**

Between 1st March and 30th June 2020, 984 referrals were made to the QEHB LP service, compared to 1020 referrals in 2019, representing a 3.5% reduction. From 2019 to 2020, referrals due to psychotic symptoms and deliberate self-harm rose by 12.8% and 14.1% respectively, whilst referrals for drug and alcohol-related causes reduced by 28.3%. A significant increase (150%) in referrals for medication or management advice was seen. Focus groups indicated that staff perceived an initial reduction in number of referrals, but an increase in the acuity of patient presentations.

Staff reported anxiety around contracting and transmitting SARS-Cov-2, exacerbated by uncertainty around patients’ COVID-19 status. In QEHB, sixty-five of the 984 referrals (7%) had a positive SARS-Cov-2 PCR swab, with the remaining 919 referrals being either negative (68%) or unknown (25%). Ninety-six percent of consultations were conducted face-to-face in QEHB. There were conflicting views amongst staff regarding whether more consultations could have been conducted remotely. Furthermore, varying perceptions of support and communication from both the physical and mental health trust were reported.

**Conclusion:**

Quantitative data indicates that COVID-19 impacted LP healthcare provision in BSMHFT. Whilst referral numbers remained similar between the equivalent period in 2019 and 2020, a change in the nature of referrals to LP at QEHB was seen. This was corroborated by qualitative data which highlighted a perceived change in acuity of referrals. These findings have been disseminated across the Trust and subsequent recommendations are being implemented during the on-going pandemic.

